# Microbiological Patterns in Periprosthetic Knee Infections over a Decade: Analysis of Resistance Patterns, Temporal Trends, and Patient Residence

**DOI:** 10.3390/antibiotics15050481

**Published:** 2026-05-09

**Authors:** Marcos González-Alonso, Alfonso Lajara-Heredia, Adrián Guerra-González, Vega Villar-Suárez, Jaime Antonio Sánchez-Lázaro

**Affiliations:** 1 Orthopaedic Surgery Department, Complejo Asistencial Universitario de León, 24008 León, Spain; 2 Department of Medicine, Surgery and Veterinary Anatomy, University of León-Universidad de León (IBIOMED), 24007 León, Spain; 3 Orthopaedic Surgery Department, Hospital of El Bierzo, 24404 Ponferrada, Spain

**Keywords:** periprosthetic joint infection, total knee arthroplasty infection, rural population, epidemiology, antimicrobial resistance, staphylococcus aureus

## Abstract

**Background:** Infection following total knee arthroplasty (TKA) is a challenging complication. Optimal empirical antibiotic therapy and surgical management hinge on up-to-date knowledge of local pathogen distribution and resistance patterns. However, few studies have examined whether geographical factors, specifically rural versus urban residence, influence the microbiology or clinical outcomes of periprosthetic joint infection (PJI) within integrated healthcare systems. The goal of this study was to assess the temporal evolution of bacterial species and antimicrobial resistance in knee PJI over an 11-year period. As a secondary objective, we wanted to evaluate the potential impact of patient residence on microbiological trends and treatment success. **Methods:** We conducted a retrospective analysis of all patients diagnosed with knee PJI who underwent surgical treatment between 2013 and 2023 at our center. Infections were classified as acute postoperative, acute hematogenous, or chronic. Patient residence was categorized as rural (<5000 inhabitants) or urban. Temporal trends were modeled using Poisson regression, and comparisons between subgroups were performed using Fisher’s exact test and Student’s t-test. **Results:** A total of 98 patients were analyzed, with 99 microorganisms identified. Gram-positive organisms predominated (72.3%), with *Staphylococcus aureus* (33.3%) and Coagulase-negative *Staphylococci* (CoNS) (29.3%) as the most frequent isolates. Resistance to vancomycin was not detected in *S. aureus* isolates. However, CoNS demonstrated high resistance to fluoroquinolones (55.2%) and rifampicin (20.7%). No significant annual shifts were observed for Gram-positive (IRR = 0.94; 95% CI: 0.86–1.03; *p* = 0.413) or Gram-negative cases (IRR = 0.75; 95% CI: 0.53–1.05; *p* = 0.086). Comparing rural versus urban populations, no differences were found in microbiological profiles (Fisher’s exact test, all *p* > 0.05). Furthermore, clinical treatment success rates were comparable (Rural 69.4% vs. Urban 63.0%, *p* = 0.500), despite a significantly higher prevalence of diabetes mellitus in rural patients (34.7% vs. 10.2%, *p* = 0.007). **Conclusions:** The microbiological landscape of knee PJI has remained stable, with no emergence of multidrug-resistant S. aureus. In our setting, standardized management protocols appeared to be equally effective regardless of patient residence. However, given the single-center nature and sample size of this study, broader multicenter validation is required before these findings can be generalized.

## 1. Introduction

Total knee arthroplasty (TKA) procedures are constantly increasing. Periprosthetic joint infection (PJI) after TKA represents a potentially devastating complication. Although they remain relatively uncommon, affecting approximately 1–3% of primary cases, with substantially higher rates following revisions [1,2,3], PJIs are associated with increased morbidity, prolonged hospitalization, higher rates of reoperation, amplified healthcare costs, and substantial detriment to patient quality of life [4,5,6].

The rise in antimicrobial resistance is a pressing global health threat that underscores the critical need for continuous epidemiological surveillance to guide effective empirical treatments [7]. A crucial factor in the persistence of these infections is the ability of microorganisms, particularly *Staphylococcus* species, to form biofilms on implant surfaces [8,9]. These complex three-dimensional structures not only facilitate evasion of the host immune system but also increase tolerance to antimicrobials, often requiring higher concentrations than those needed for planktonic bacteria [7,10]. Consequently, the standard treatment of deep knee PJI demands a combination of surgical debridement and prolonged, targeted antibiotic therapy, making the correct initial identification of the pathogen one of several critical factors for treatment success, alongside surgical technique and host optimization [11,12]. Yet, culture-negative PJIs remain frequent, with published prevalence rates ranging from about 5% to 22% [13,14,15]. In these cases, empirical broad-spectrum antibiotics are initiated post-debridement or during two-stage exchange procedures, often without precise microbiological guidance, increasing the risk of suboptimal treatment and antibiotic-related adverse effects [16]. Therefore, local epidemiological data on PJIs in terms of pathogen distribution and susceptibility patterns serve as the foundation upon which empirical clinical protocols are built, ensuring they are tailored to the actual local microbial landscape [17,18].

Although prior studies have described microbial profiles in PJI, most derive from urban centers [12,19,20]. Our center is a tertiary referral hospital that receives patients from mid-sized cities and rural areas. Demographic factors such as community size or rural area residency have not yet been studied in the context of knee PJIs. While differences linked to rurality are not well-documented, analyzing this variable in a population characterized by aging and dispersion represents an exploratory approach to identify potential variations in microbiological profiles. Relying solely on international guidelines without validating them against local data carries the risk of empirical treatment failure or, conversely, the overuse of broad-spectrum antibiotics that promote further resistance.

The purpose of this study is to characterize the microorganisms responsible for deep infections following TKA at our institution over an 11-year period and to evaluate temporal trends in pathogen distribution and resistance patterns. The secondary aim of this study was to assess the potential impact of rurality on these microbiological profiles.

## 2. Results

### 2.1. Demographics and Clinical Presentation

Over the 11-year study period, 98 patients underwent surgical treatment for infected TKA. The cohort was evenly distributed, with 49 patients (50.0%) residing in urban areas and 49 (50.0%) in rural areas (Table 1). No significant differences were observed between groups regarding sex, age, or length of hospital stay. Among comorbidities, univariate analysis showed that only diabetes mellitus was significantly more prevalent in the rural area (34.7% vs. 10.2%, *p* = 0.007); however, these variables were not adjusted for potential confounders.

Regarding clinical presentation, joint effusion was the most frequent sign, observed in 42.9% of patients, followed by the presence of a sinus tract or wound dehiscence in 26.5%. Based on the temporal classification of the infection, 43 cases (43.9%) were acute postoperative infections, 37 (37.8%) were acute hematogenous, and 18 (18.4%) were chronic infections.

### 2.2. General Microbiology and Temporal Trends

**Figure 1:**
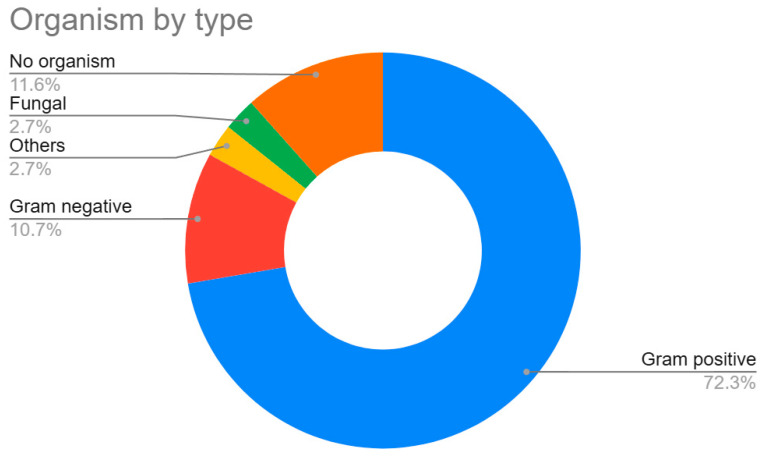
Organism by type, based on Gram stain, fungal growth, or no organism identified (N = 98).

A total of 99 microorganisms were isolated during the study period with 12 (12.2%) patients presenting polymicrobial infections. Gram-positive bacteria accounted for 72.3% of isolates, followed by Gram-negative (10.7%), other bacterial species such as *Mycobacterium* spp. and *Mycoplasma* spp. (2.7%), and fungal organisms (2.7%). In 11.6% of samples, no organism was identified (Figure 1).

**Figure 2:**
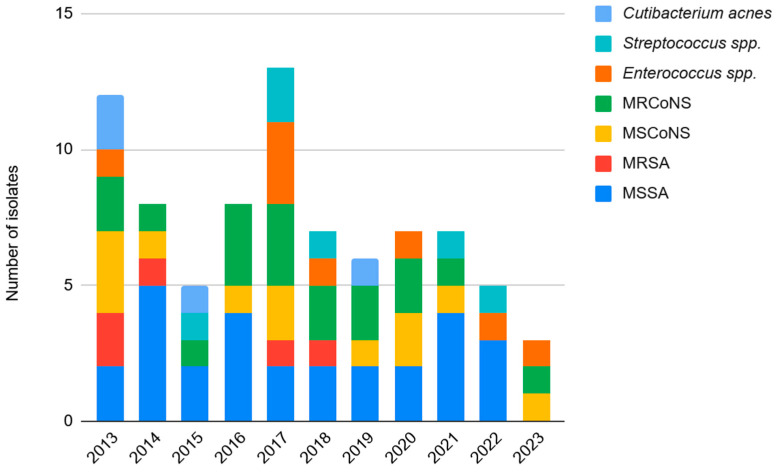
Temporal distribution of Gram-positive bacteria and fungal organisms causing PJI (2013–2023). Abbreviations: MSSA: Methicillin-sensitive *Staphylococcus aureus*; MRSA: Methicillin-resistant *Staphylococcus aureus*; MSCoNS: Methicillin-sensitive Coagulase-Negative *Staphylococci*; MRCoNS: Methicillin-resistant Coagulase-Negative *Staphylococci*.

**Figure 3:**
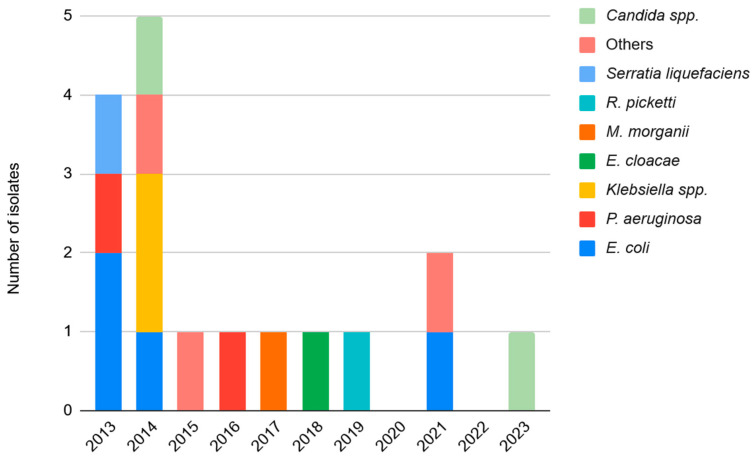
Temporal distribution of Gram-negative bacteria and fungal organisms causing PJI (2013–2023).

Annual infection rates for specific Gram-positive species are detailed in Figure 2, while temporal trends of Gram-negative, fungal, and other organisms are shown in Figure 3. Poisson regression models adjusted for annual total knee PJI cases revealed no statistically significant temporal trends in the incidence of Gram-positive (IRR = 0.94; 95% CI: 0.86–1.03; *p* = 0.413), Gram-negative (IRR = 0.75; 95% CI: 0.53–1.05; *p* = 0.086), fungal (IRR = 1.05; 95% CI: 0.67–1.63; *p* = 0.459) or culture-negative cases (IRR = 0.88; 95% CI: 0.58–1.34; *p* = 0.577) over the 11-year study period. Notably, Gram-negative infections showed a decreasing trend approaching statistical significance. Analysis was performed as well for all species with a Bonferroni correction for multiple comparisons to minimize the risk of Type I errors given the multiple species comparisons and there were no significant differences among all species causing PJI in TKA (all adjusted *p* > 0.05). Similarly, no significant changes were observed in the frequency of specific species such as *S. aureus* or Coagulase-negative *Staphylococci* (CoNS).

### 2.3. Rural vs. Urban Comparison

**Figure 4:**
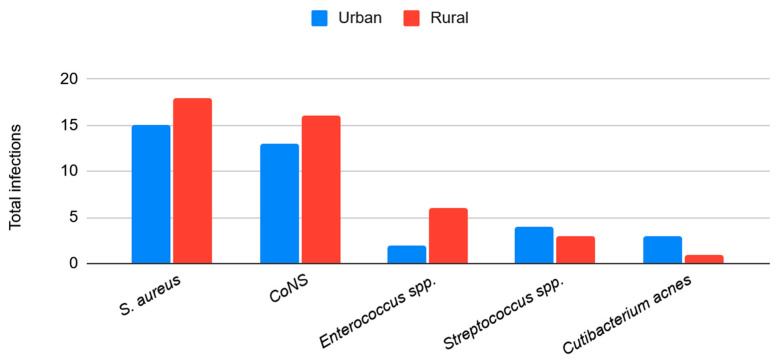
Total Gram-positive infections during the study period by area of residency (urban vs. rural). Data shown as absolute numbers per year. Abbreviation: CoNS: Coagulase-negative *Staphylococci*.

**Figure 5:**
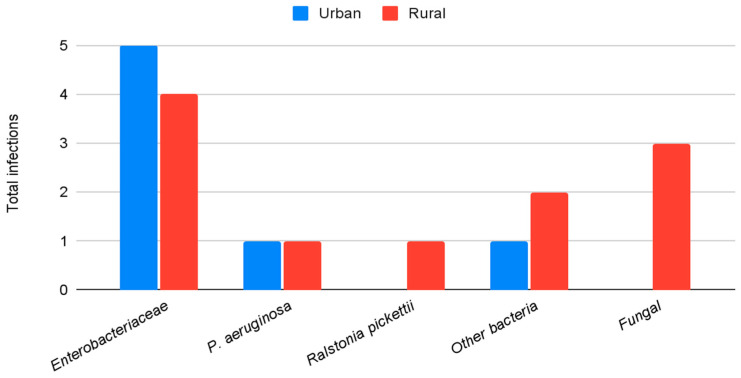
Total Gram-negative, other bacteria and fungal infections during the study period by area of residency (urban vs. rural). Data shown as absolute numbers per year.

Microbiological profiles were similar between study groups. No statistically significant differences were observed in the distribution of Gram-positive (Figure 4) or Gram-negative (Figure 5) organisms between rural and urban areas. However, all fungal infections (*n* = 3) occurred in patients from rural areas. Regarding polymicrobial infections (*n* = 12), 66.7% were identified in the rural population compared to 33.3% in the urban population.

### 2.4. Antimicrobial Susceptibility

Among *S. aureus* isolates (*n* = 33), 15.2% were methicillin-resistant (MRSA). Resistance to rifampicin and levofloxacin was 21.2% and 33.3%, respectively. No isolates showed resistance to vancomycin or daptomycin. CoNS isolates (*n* = 29) demonstrated higher resistance rates, particularly to methicillin (48.3%), fluoroquinolones (55.2%), and gentamicin (41.4%).

Regarding Gram-negative bacteria (*n* = 12), resistance to the tested antibiotics was rare although these results should be interpreted with caution due to the limited sample size.

No statistically significant differences in resistance patterns were found between rural and urban areas across any species–antibiotic combinations (Fisher’s exact test, all *p* > 0.05). Resistance profiles of all isolated microorganisms are summarized in Table 2 and Table 3.

## 3. Discussion

This 11-year retrospective analysis of PJI in TKA patients reveals a remarkably stable microbiological profile over time, with Gram-positive cocci continuing to predominate. MSSA and CoNS were the most frequently isolated species, consistent with previous reports [12,19,20]. No significant changes in the frequency of specific organisms or in antimicrobial resistance patterns were observed throughout the study period, including among rural versus urban populations.

These findings align with earlier multicenter cohorts and national registry data that demonstrate the persistent dominance of Gram-positive bacteria in PJIs [21,22]. Our stability in pathogen distribution may reflect consistent surgical practices, antibiotic prophylaxis, and diagnostic protocols across the years. The absence of resistance to vancomycin and daptomycin among *S. aureus* isolates is encouraging, although these findings should be interpreted cautiously given the limited sample size. Nevertheless, they support the continued use of glycopeptides and lipopeptides as reliable empirical options in our setting.

In contrast, the high prevalence of resistance to fluoroquinolones (55.2%) and rifampicin (20.7%) among CoNS is concerning. These agents are often used in biofilm-active combination therapy [23]. Resistance patterns such as these underscore the need to confirm susceptibility before initiating rifampicin-based regimens and highlight the need of adjuvant antibiofilm measures such as intraoperative irrigation with antiseptic solutions [24]. Our findings are consistent with other European series reporting increasing CoNS resistance, likely influenced by chronic infection cases and prior antibiotic exposure [8,11].

In contrast, Gram-negative isolates were infrequent (10.7%) and exhibited low rates of resistance to commonly used beta-lactams. While susceptibility to cefepime and piperacillin-tazobactam remains high, the identification of isolated cases resistant to imipenem or ciprofloxacin warrants vigilance. These results support current protocols for empirical coverage in early postoperative or hematogenous infections, particularly those involving urinary tract sources [25].

Our culture-negative rate of 11.6% in the cohort falls within the 5–22% range reported in the literature [15,26,27]. However, this figure should be contextualized within our institutional protocol, where tissue cultures are discarded after 10 days. Given that organisms such as *Cutibacterium acnes* may require up to 14 days for optimal growth [28,29], it is possible that a proportion of our culture-negative cases represents under-detected fastidious organisms. This highlights a clear opportunity for diagnostic improvement through prolonged incubation periods or the integration of molecular tools like multiplex PCR [30,31].

The observed microbiological homogeneity between rural and urban populations suggests that geographical dispersion does not impact PJI etiology in our setting. To our knowledge, this is one of the first studies to assess the impact of rurality on PJI microbiology in a healthcare system with universal access. Interestingly, geographical setting, characterized by aging and population dispersion, had no measurable impact on pathogen type or resistance patterns in our sample. Although diabetes mellitus was significantly more prevalent in the rural cohort (34.7% vs. 10.2%), this did not translate into worse microbiological profiles or inferior outcomes. The finding that rural residence did not influence microbiological profiles despite a higher burden of diabetes suggests that in healthcare systems with universal coverage, geographical dispersion does not necessarily limit access to timely and standardized surgical care. Furthermore, it implies that the commensal flora of patients in our catchment area remains relatively homogeneous, regardless of the population density of their primary residence. This finding suggests that standardized care pathways, as also observed in US Veteran populations [32], effectively mitigate the influence of baseline demographic disparities on PJI characteristics.

This study has several strengths. It spans more than an entire decade with consistent diagnostic and therapeutic protocols in a tertiary referral hospital, providing a longitudinal view of microbiological stability. The inclusion of patients from both rural and urban areas enhances the external validity of our findings.

However, certain limitations must be acknowledged. First, by including only surgically treated PJIs, we may have introduced a selection bias, potentially excluding less severe cases managed solely with suppressive antibiotic therapy. Second, its retrospective design limits control over pre-analytical variables, such as the exact timing of antibiotic initiation. Third, the relatively low annual incidence limits our statistical power to detect small differences in rare pathogens or subtle temporal shifts.

## 4. Materials and Methods

### 4.1. Study Design and Participants

We conducted a retrospective cohort study of all patients diagnosed with knee PJI who underwent surgical treatment between 1 January 2013, and 31 December 2023, at a tertiary referral hospital serving a mixed urban and rural population. The study was performed in accordance with the Declaration of Helsinki and was approved by the local Ethics Committee (code: 23126). Informed consent for participation was obtained from all subjects involved in the study.

We followed a consecutive sampling procedure. Patients were included if they met the following criteria: (1) diagnosis of knee PJI according to the study definitions; (2) underwent surgical intervention for infection (debridement, antibiotics, and implant retention [DAIR], one-stage or two-stage revision, or arthrodesis); (3) had a minimum follow-up of one year after treatment completion; and (4) had complete medical records, including operative reports and microbiological data. Patients were excluded if they underwent revision surgery for aseptic causes or if essential clinical or microbiological data were missing.

### 4.2. Definitions and Diagnosis

Knee PJI was defined according to the 2018 International Consensus Meeting criteria [26]. Cases diagnosed prior to 2018 were retrospectively re-evaluated; those not meeting the 2018 criteria but treated as PJI based on the Musculoskeletal Infection Society (MSIS) criteria [33] or a strong combination of clinical suspicion, elevated inflammatory markers, and positive cultures, were also included to ensure cohort consistency. Culture-negative cases were included if purulence was observed intraoperatively or if a sinus tract communicating with the prosthesis was present. Treatment success was defined as infection resolution after first surgical procedure and limited antibiotic treatment without further surgeries.

Infections were classified based on temporal presentation and symptom duration following the criteria proposed by Zimmerli et al. [34]. Acute postoperative PJI was defined as an infection occurring within three months of the index surgery with symptoms lasting less than three weeks. Acute hematogenous PJI was defined as an acute onset of symptoms (<3 weeks) in a previously asymptomatic prosthetic joint. All other cases, including those with symptoms persisting for more than three weeks or presenting beyond the early postoperative period without acute onset, were classified as chronic PJI.

Patient residence was categorized into two groups based on the population size of their municipality, using the most recent data from the Spanish National Statistics Institute [35]. Following the national classification for rurality (Royal Decree 137/1984), patients residing in municipalities with fewer than 5000 inhabitants were classified as “rural,” while those in municipalities with 5000 or more inhabitants were classified as “urban.”

### 4.3. Data Collection and Microbiology

Data were extracted from electronic medical records, including patient demographics (age, sex, area of residence) and specific comorbidities relevant to infection risk: diabetes mellitus, chronic kidney disease, rheumatoid arthritis, hepatopathy, and chronic obstructive pulmonary disease. Surgical details and microbiological results were also recorded.

Intraoperative microbiological sampling was performed, obtaining a range of two to seven deep tissue specimens from the bone–implant interface and periprosthetic soft tissues. Synovial fluid was also cultured when available. Identification of microorganisms was performed using the VITEK^®^ 2 automated system (bioMérieux, Marcy-l’Étoile, France) and, in recent years, MALDI-TOF VITEK MS (Matrix-Assisted Laser Desorption/Ionization-Time of Flight Mass Spectrometry) (bioMérieux, Marcy-l’Étoile, France). These automated methods were complemented by conventional manual processing and biochemical assays (e.g., catalase and coagulase tests) for specific isolate characterization.

Growth detection was monitored using the BD BACTEC™ automated system (Becton, Dickinson and Company, Sparks, MD, USA) for all blood and fluid cultures. Aerobic and anaerobic cultures were maintained for a standard duration of 10 days throughout the study period. Antibiotic Susceptibility Testing (AST) was primarily performed by determining the Minimum Inhibitory Concentration (MIC) using the VITEK^®^ 2 automated system (bioMérieux, Marcy-l’Étoile, France). For specific isolates or antibiotics where automated testing was not available or required clinical confirmation, AST was supplemented by disk diffusion (Kirby-Bauer) or gradient diffusion strips (E-test, bioMérieux, Marcy-l’Étoile, France). All AST results were interpreted according to the European Committee on Antimicrobial Susceptibility Testing (EUCAST) breakpoints current at the time of isolation [36]. Polymicrobial infections were defined as the isolation of two or more distinct microorganisms.

### 4.4. Statistical Analysis

Continuous variables were described as means with standard deviations (SD) or medians with interquartile ranges (IQR), depending on their distribution. Categorical variables were presented as frequencies and percentages. Differences between rural and urban groups were analyzed using the independent *t*-test or Mann–Whitney U test for continuous variables, and the Chi-square test or Fisher’s exact test for categorical variables, as appropriate.

To assess temporal trends in the incidence of specific pathogens and resistance patterns over the 11-year period, we calculated the annual rate of PJI for each organism and analyzed trends using Poisson regression models, reporting Incidence Rate Ratios (IRR) with 95% Confidence Intervals (CI). Statistical significance was set at *p* < 0.05. Bonferroni correction was applied for multiple comparisons to strictly control for Type I errors given the number of species analyzed. All statistical analyses were performed using IBM SPSS Statistics for Windows, Version 28.0 (IBM Corp., Armonk, NY, USA).

## 5. Conclusions

The microbiological landscape of knee PJI in our setting has remained stable over the past decade, with *S. aureus* and *CoNS* as the predominant pathogens. No significant temporal shifts were observed in species distribution or antimicrobial resistance patterns. Resistance to vancomycin was not detected in *S. aureus* isolates and remained low across other species.

Comparing our rural and urban populations, we found no significant differences in microbiological profiles or resistance patterns. Furthermore, clinical outcomes were comparable between groups, with similar treatment success rates despite a higher prevalence of diabetes mellitus in rural patients.

These findings suggest that there is no microbiological or clinical justification for differentiating empirical antibiotic or surgical protocols based on patient residence in our area at this time. Standardized management remains effective and should continue to be guided by local epidemiology rather than geographical factors. However, given the single-center nature and sample size of this study, broader multicenter validation is required before these findings can be generalized to other centers.

## Figures and Tables

**Table 1:** Demographic and clinical characteristics of patients with PJI, stratified by area of residence (urban vs. rural).

	Urban	Rural	Statistical *p*-Value
Group size (N = 98)	49 (50.00%)	49 (50.00%)	
Men	18 (36.73%)	19 (38.78%)	0.834
Women	31 (63.27%)	30 (61.22%)	
Mean age (SD)	74.41 (8.12)	72.51(10.41)	0.316
Mean length of stay- Days (SD)	24.82 (19.75)	32.76 (33.06)	0.153
Comorbidities:			
COPD	3 (6.12%)	6 (12.24%)	0.486
Hepatopathy	2 (4.08%)	3 (6.12%)	1.000
Rheumatoid arthritis	4 (8.16%)	4 (8.16%)	1.000
CKD	7 (14.29%)	7 (14.29%)	1.000
Diabetes mellitus	5 (10.20%)	17 (34.69%)	0.007

Data are presented as *n* (%) for categorical variables and mean (SD) for continuous variables. *p*-values were calculated using Fisher’s exact test for categorical variables, and Student’s *t*-test for continuous variables (age and length of stay). Abbreviations: SD: standard deviation; COPD: Chronic Obstructive Pulmonary Disease; CKD: Chronic Kidney Disease.

**Table 2:** Gram-positive bacterial resistance pattern by area of residency.

	Antibiotic Resistance		Total	Fisher’s Exact Test *p*-Value
	Urban	Rural		
***S. aureus***				
Methicillin	2/15 (13.33%)	3/18 (16.67%)	5/33 (15.15%)	1.00
Rifampicin	3/15 (20.00%)	4/18 (22.22%)	7/33 (21.21%)	1.00
Vancomycin	0/15 (0.00%)	0/18 (0.00%)	0/33 (0.00%)	1.00
Teicoplanin	1/15 (6.67%)	2/18 (11.11%)	3/33 (9.09%)	1.00
Levofloxacin	6/15 (40.00%)	5/18 (27.78%)	11/33 (33.33%)	0.51
Gentamicin	1/15 (6.67%)	4/18 (22.22%)	5/33 (15.15%)	0.41
Clindamycin	4/15 (26.67%)	3/18 (16.67%)	7/33 (21.21%)	0.51
Daptomycin	0/15 (0.00%)	0/15 (0.00%)	0/33 (0.00%)	1.00
***CoNS***				
Methicillin	9/13 (69.23%)	5/16 (31.25%)	14/29 (48.28%)	0.14
Rifampicin	2/13 (15.38%)	4/16 (25.00%)	6/29 (20.69%)	0.71
Vancomycin	0/13 (0.00%)	1/16 (6.25%)	1/29 (3.45%)	1.00
Teicoplanin	1/13 (7.69%)	2/16 (12.50%)	3/29 (10.34%)	1.00
Levofloxacin	8/13 (61.54%)	8/16 (50.00%)	16/29 (55.17%)	0.71
Gentamicin	7/13 (53.85%)	5/16 (31.25%)	12/29 (41.38%)	0.72
Clindamycin	4/13 (30.77%)	6/16 (37.50%)	10/29 (34.48%)	0.72
Daptomycin	0/13 (0.00%)	0/16 (0.00%)	0/29 (0.00%)	1.00
***Enterococcus*** **spp.**				
Vancomycin	0/2 (0.00%)	1/6 (16.67%)	1/8 (12.50%)	1.000
Gentamicin	2/2 (100%)	3/6 (50.00%)	5/8 (62.50%)	0.540
Levofloxacin	1/2 (50.00%)	2/6 (33.33%)	3/8 (37.50%)	1.000
Linezolid	2/2 (100%)	4/6 (66.67%)	6/8 (75.00%)	0.539
Amoxicillin/clavulanate	0/2 (0.00%)	0/6 (0.00%)	0/8 (0.00%)	1.000
Daptomycin	0/2 (0.00%)	0/6 (0.00%)	0/8 (0.00%)	1.000
***Streptococcus*** **spp.**				
Rifampicin	0/4 (0.00%)	0/3 (0.00%)	0/7 (0.00%)	1.000
Clindamycin	1/4 (25.00%)	2/3 (66.67%)	3/7 (42.86%)	0.494
Vancomycin	0/4 (0.00%)	0/3 (0.00%)	0/7 (0.00%)	1.000
Amoxicillin/clavulanate	0/4 (0.00%)	0/3 (0.00%)	0/7 (0.00%)	1.000
***Cutibacterium acnes***				
Clindamycin	0/3 (0.00%)	0/1 (0.00%)	0/4 (0.00%)	1.000
Vancomycin	0/3 (0.00%)	0/1 (0.00%)	0/4 (0.00%)	1.000
Rifampicin	0/3 (0.00%)	0/1 (0.00%)	0/4 (0.00%)	1.000
Amoxicillin/clavulanate	0/3 (0.00%)	0/1 (0.00%)	0/4 (0.00%)	1.000

Abbreviations: CoNS: Coagulase-negative *Staphylococci.* Resistance rates are expressed as the ratio of resistant strains to the total number of unique isolates, regardless of the number of patients.

**Table 3:** Most frequent Gram-negative bacteria resistance pattern by area of residency.

	Antibiotic Resistance		Total	Fisher’s Exact Test *p*-Value
	Urban	Rural		
***Enterobacteriaceae***				
Imipenem	0/5 (0.00%)	1/4 (25.00%)	1/9 (11.11%)	1.00
Piperacillin/tazobactam	0/5 (0.00%)	0/4 (0.00%)	0/9 (0.00%)	1.00
Cefepime	0/5 (0.00%)	0/4 (0.00%)	0/9 (0.00%)	1.00
Cotrimoxazole	0/5 (0.00%)	0/4 (0.00%)	0/9 (0.00%)	1.00
Ciprofloxacin	0/5 (0.00%)	0/4 (0.00%)	0/9 (0.00%)	1.00
***Pseudomonas***				
Ciprofloxacin	0/1 (0.00%)	1/1 (100%)	1/2 (50.00%)	1.00
Gentamicin	0/1 (0.00%)	0/1 (0.00%)	0/2 (0.00%)	1.00
Imipenem	0/1 (0.00%)	0/1 (0.00%)	0/2 (0.00%)	1.00

Resistance rates are expressed as the ratio of resistant strains to the total number of unique isolates, regardless of the number of patients.

## Data Availability

The original contributions presented in this study are included in the article. Further inquiries can be directed to the corresponding author.
